# Malaria Mosquitoes Attracted by Fatal Fungus

**DOI:** 10.1371/journal.pone.0062632

**Published:** 2013-05-01

**Authors:** Justin George, Nina E. Jenkins, Simon Blanford, Matthew B. Thomas, Thomas C. Baker

**Affiliations:** 1 Department of Entomology, Pennsylvania State University, University Park, Pennsylvania, United States of America; 2 Center for Infectious Disease Dynamics, Pennsylvania State University, University Park, Pennsylvania, United States of America; Centro de Investigación y de Estudios Avanzados, Mexico

## Abstract

Insect-killing fungi such as *Beauveria bassiana* are being evaluated as possible active ingredients for use in novel biopesticides against mosquito vectors that transmit malaria. Fungal pathogens infect through contact and so applications of spores to surfaces such as walls, nets, or other resting sites provide possible routes to infect mosquitoes in and around domestic dwellings. However, some insects can detect and actively avoid fungal spores to reduce infection risk. If true for mosquitoes, such behavior could render the biopesticide approach ineffective. Here we find that the spores of *B. bassiana* are highly attractive to females of *Anopheles stephensi*, a major anopheline mosquito vector of human malaria in Asia. We further find that *An. stephensi* females are preferentially attracted to dead and dying caterpillars infected with *B. bassiana,* landing on them and subsequently becoming infected with the fungus. Females are also preferentially attracted to cloth sprayed with oil-formulated *B. bassiana* spores, with 95% of the attracted females becoming infected after a one-minute visit on the cloth. This is the first report of an insect being attracted to a lethal fungal pathogen. The exact mechanisms involved in this behavior remain unclear. Nonetheless, our results indicate that biopesticidal formulations comprising *B. bassiana* spores will be conducive to attraction and on-source visitation by malaria vectors.

## Introduction

Malaria remains the most devastating of insect-vectored diseases. Successful control of malaria mosquitoes (species in the genus *Anopheles*), is a proven, effective way to reduce transmission of malaria. Motivated by mounting problems of resistance to conventional chemical insecticides [1], recent years have seen growing interest in the potential for developing novel biopesticides comprising insect-killing fungi, such as *Beauveria bassiana* and *Metarhizium anisopliae*, for control of adult malaria mosquitoes. These entomopathogenic fungal spores can infect and kill the mosquitoes in 1–2 weeks depending on the dosage, mosquito exposure duration, and the viability and virulence of the fungal strain [2]–[5]. Entomopathogenic fungi have also been tested for their efficacy against other blood feeding insects such as ticks [6], tsetse flies [7], triatomid bugs [8] and bedbugs [9].

Although fungal pathogens kill more slowly than conventional chemical insecticides, they can still contribute to malaria control by shortening mosquito life span and reducing the number of mosquitoes that are old enough to successfully incubate and transmit the malaria parasite [10]–[12]. In principle, slow-death mode of action of fungi also reduces the possible buildup of resistance by mosquitoes to fungal biopesticides [10]–[12]. In addition to the lethal effects, fungal pathogens can cause a range of pre- or sub-lethal effects in mosquitoes and other arthropods, including the reduction in feeding [13], flight performance [5], [14], predator avoidance [15] and alteration in development [16]. Reduction in feeding or host-finding as a result of fungal infection has been shown in *Anopheles*, *Culex* and *Aedes* mosquitoes [2], [5], [17]–[19]. Our recent work [18] had shown that the pre-lethal effects of infection with *B. bassiana* fungal spores include a reduced ability of female *An. stephensi* to respond to host-related odor cues by flying upwind, as well as a suppression of olfactory receptor neurons tuned to 1-octen-3-ol, a mammalian odorant known to attract biting flies, including mosquitoes [20]–[22].

To infect mosquitoes in the field requires physical contact with fungal spores sprayed on surfaces such as walls, nets, or other resting targets in and around domestic dwellings. However, evidence from a number of systems suggests that some insects can detect and actively avoid fungal spores to reduce infection risk [23]. Avoidance of a lethal fungus makes adaptive sense. If true for mosquitoes, such behavior could dramatically reduce efficacy of the biopesticide approach.

In order to determine the behavioral response of mosquitoes to *B. bassiana* spores we performed a suite of Y-tube olfactometer behavioral bioassays using females of the Asian malaria vector, *Anopheles stephensi*. We tested the attraction of dry spore powder pitting *B. bassiana* against both clean air and other fungi: a less virulent strain of the entomopathogen, *Metarhizium anisopliae*; and an opportunistic saprophyte, *Penicillium* sp. Contrary to expectations, *An. stephensi* females were preferentially attracted to the arm of the Y-tube containing lethal *B. bassiana* spores. Further olfactometer assays showed that the females became infected with *B. bassiana* after visiting dried *B. bassiana* spores or else after visiting the sporulating cadavers of caterpillars that had been infected with *B. bassiana*. Female *An. stephensi* were also preferentially attracted to cloth sprayed with a biopesticidal formulation of *B. bassiana* spores, with 95% of the females visiting the cloth becoming infected.

## Results

### Attraction of *An. stephensi* Females to Dried Spores of Various Fungal Species

**Figure 1:**
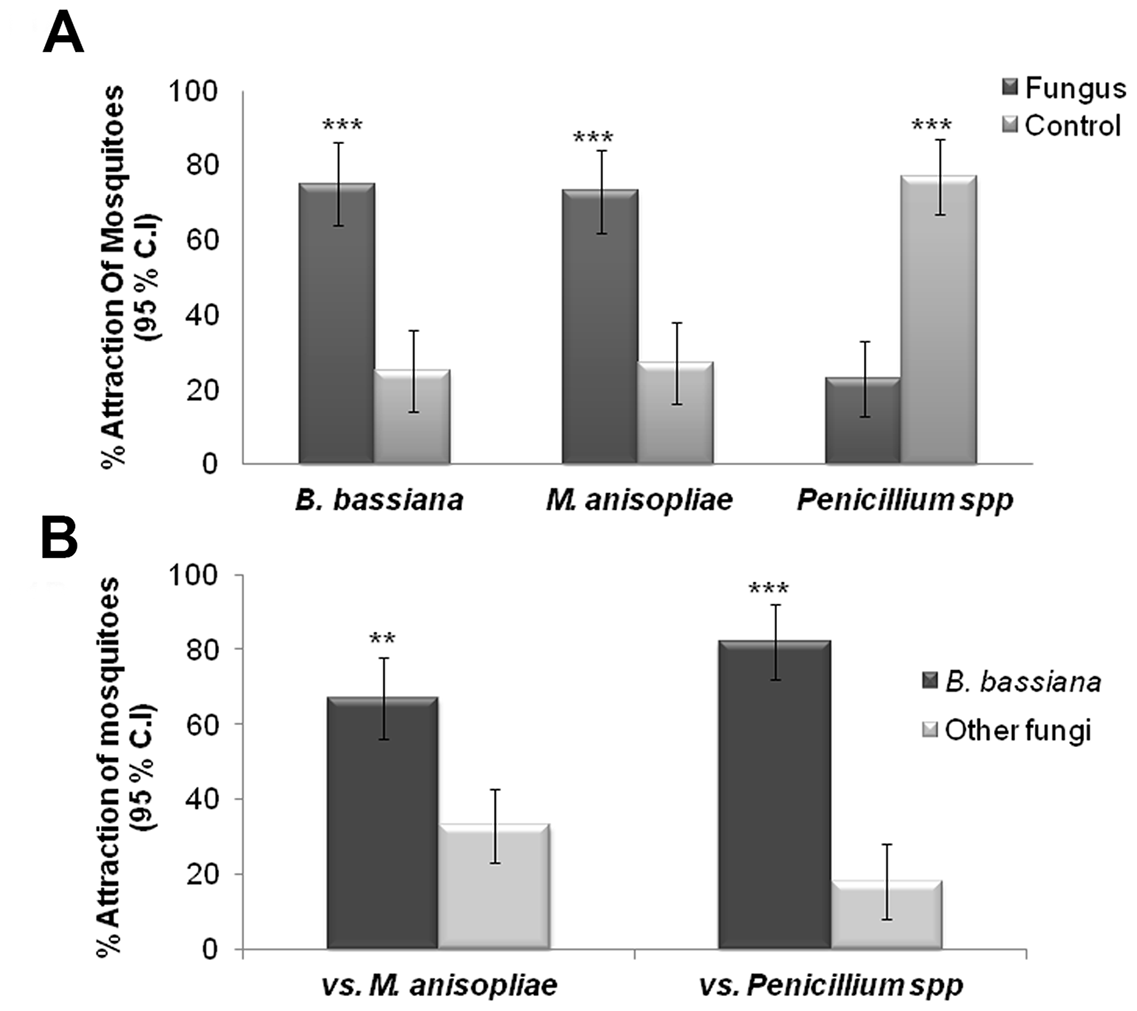
Dried fungal spores of *B. bassiana* and *M. anisopliae* are attractive to female *An. stephensi*, but spores of *B. bassiana* are more attractive than those of *M. anisopliae* or *Penicillium* spp. **A)** Percentage of females attracted to 50 mg of dried spores of *B. bassiana, M. anisopliae,* or *Penicillium* spp. placed on a filter paper disc (dark bars) versus a blank disc (light bars) (N = 60 females in each comparison); **B)** Percentage of females attracted to the arm containing *B. bassiana* spores (dark bars) compared to the arm containing spores of either *M. anisopliae* or *Penicillium* (light bars) (N = 60 females in each comparison). Brackets denote ±95% binomial confidence intervals (C.I.). Asterisks indicate significant differences between attraction to treatment choice-pairs (Chi-square 2×2 test of independence; **P≤0.01; ***P≤0.001).

We performed behavioral bioassays using a Y-tube olfactometer that was sufficiently long (70 cm) and having a large-enough diameter (6 cm) such that the females could fly upwind to the test stimuli at either choice-arm of the setup. We first tested, one at a time, 50 mg of dried fungal spores of several species placed on their own filter paper discs, and found that female *An. stephensi* were attracted strongly to the spores of *B. bassiana* as well as to those of *M. anisopliae* (Fig. 1A). Females flew upwind in the Y-tubes and landed on or near the fungal spores, often exhibiting probing behaviors. They showed a significantly higher level of attraction (75% and 73%, respectively) to spores of *B. bassiana* (χ^2^ = 30, d.f = 1, P<0.0001) and *M. anisopliae* (χ^2^ = 13.07, d.f = 1, P = 0.0003) compared to the controls (25% and 23%, respectively) (Fig. 1A). Females showed significant avoidance of Y-tube arm containing *Penicillium* spores, with 76% choosing the control arm (χ^2^ = 17.06, d.f = 1, P<0.0001) (Fig. 1A). We next pitted *B. bassiana* spores against the spores of each of the other species to determine *B. bassiana*’s relative attractiveness to *An. stephensi* females. Females exhibited a significant preference of attraction to the spores of *B. bassiana* (67% and 82%, respectively) compared to spores of *M. anisopliae* (χ^2^ = 6.67, d.f = 1, P = 0.009) and *Penicillium spp* (χ^2^ = 21.6, d.f = 1, P<0.0001) (Fig. 1B).

### Attraction of *An. stephensi* Females to Sporulating Insect Cadavers

**Figure 2:**
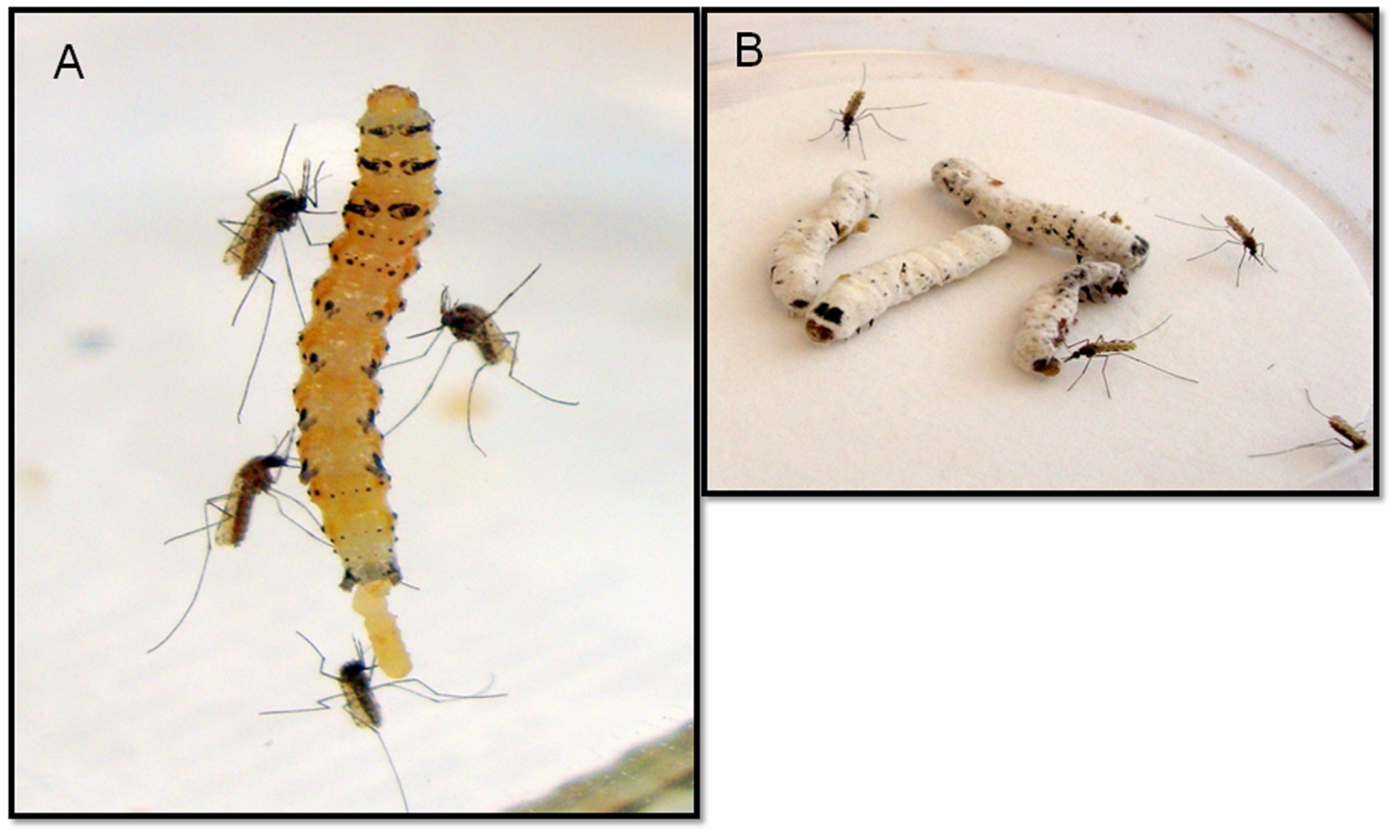
Photographs of typical *An. stephensi* females when released in groups in the presence of either live (A) or dead-sporulating (B) *H. subflexa* caterpillars. Following attraction to the caterpillars, the females land and probe them, sometimes engorging on caterpillar haemolymph (unpublished data). **A**) A healthy, uninfected *H. subflexa* 4^th^-instar caterpillar. **B**) Several dead, sporulating *H. subflexa* caterpillars that had attracted *An. stephensi* females.

**Figure 3:**
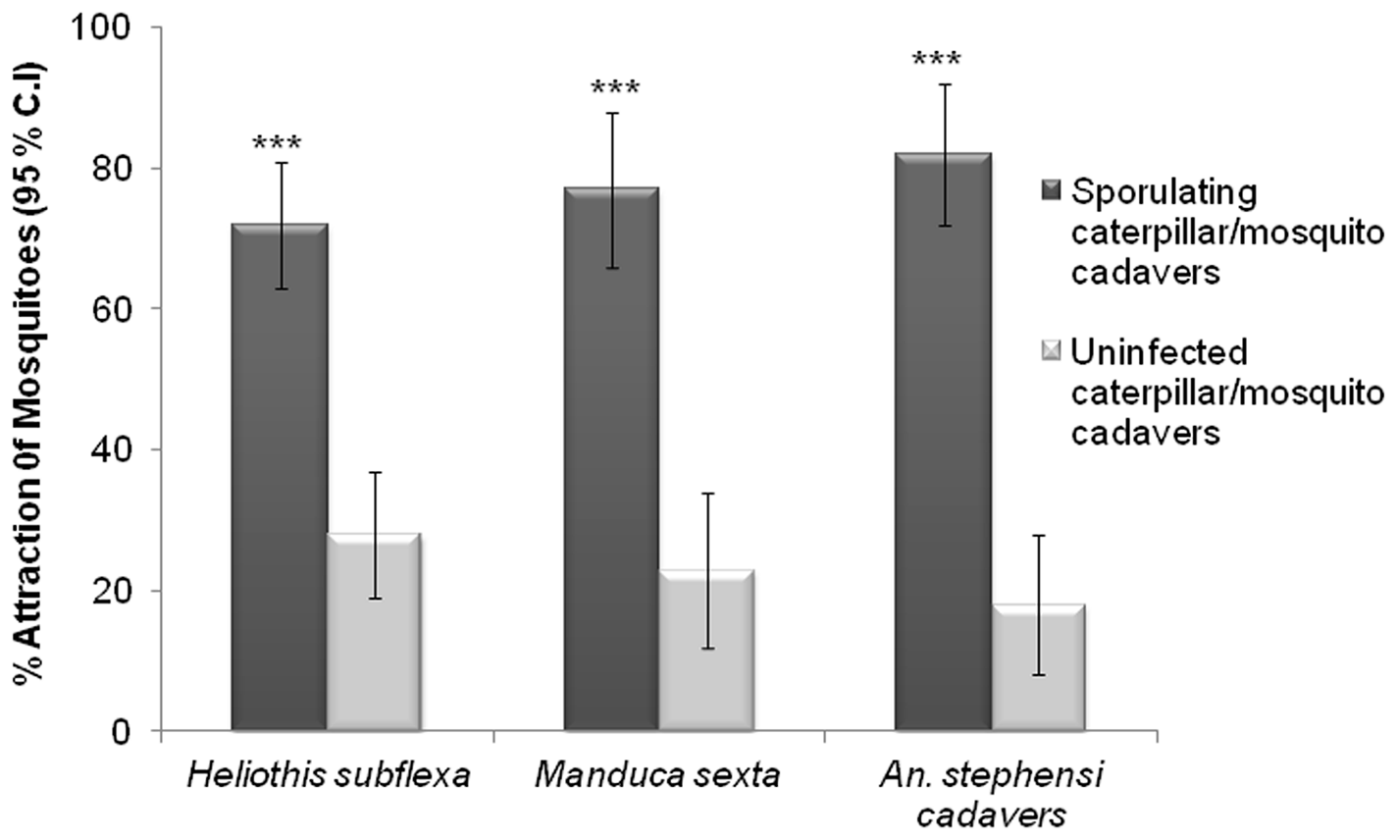
*B. bassiana*-sporulating insect cadavers are more attractive to *An. stephensi* females than uninfected cadavers. Percentage (dark bars) of individually tested *An. stephensi* females that flew upwind in the Y-tube in response to sporulating cadavers of: (left) *H. subflexa*; (center) *M. sexta*: and (right) *An. stephensi* females that had died following *B. bassiana* infection. Light bars indicate attraction to the alternate Y-tube arm containing uninfected dead caterpillars or mosquitoes. Brackets denote ±95% binomial confidence intervals (C.I.). Asterisks indicate significant differences between attraction to treatment choice-pairs (Chi-square 2×2 test of independence; ***P≤0.001; N = 60).

Because *B. bassiana* is known to cause mortality in a range of insect taxa [24]–[27] (i.e. mosquitoes might well encounter infected insects in the field), we then sought to determine the ability of *B. bassiana*-killed insect cadavers to attract *An. stephensi* females. Caterpillars have been known since the 1960 s to be among the several types of soft-bodied insect larvae that mosquitoes can and will feed upon [28]. However, not until recently have further such studies been conducted, one of which found that mosquito-feeding on caterpillars can be detrimental to lepidopteran development and adult fertility and fecundity [29]. In ongoing studies in our lab, we already had determined that *An. stephensi* females are highly attracted to healthy caterpillars of two moth species, *Manduca sexta* and *Heliothis subflexa* (unpublished data) (Fig. 2A). We thus decided to purposely infect *M. sexta* and *H. subflexa* caterpillars with *B. bassiana* to determine the affects of the fungal infection on *An. stephensi* female attraction. Caterpillars experiencing *B. bassiana* infection only show visible signs of infection several days after their death, when *B. bassiana* spores appear on the exterior of their cadavers. We found that the sporulating caterpillar cadavers (Fig. 2B) of these two species that had died following *B. bassiana* infection were significantly more attractive to *An. stephensi* females than were uninfected caterpillar cadavers (Fig. 3). The sporulating cadavers of *H. subflexa* caused 72% of released females to fly upwind to them compared to 28% flying into the arm containing uninfected cadavers (χ^2^ = 17.7, d.f = 1, P<0.0001**)** (Fig. 3, Left). Sporulating *M. sexta* cadavers caused 77% of the released females to fly upwind and land on or near them, picking up spores in the process (see below). Only 23% of the females flew to the uninfected caterpillar cadaver arm (χ^2^ = 17.06, d.f = 1, P<0.0001) (Fig. 3, Center). Furthermore, when we took the sporulating cadavers of *An. stephensi* females themselves that had been infected with *B. bassiana*, these were more attractive to *An. stephensi* females (82%) than uninfected *An. stephensi* cadavers in the alternate arm of the Y-tube (18%) (χ^2^ = 24.06, d.f = 1, P<0.0001) (Fig. 3, Right).

### Sporulation Assays: Infection of *An. stephensi* Females Following Attraction to, and Contact with, Fungal Spores

**Figure 4:**
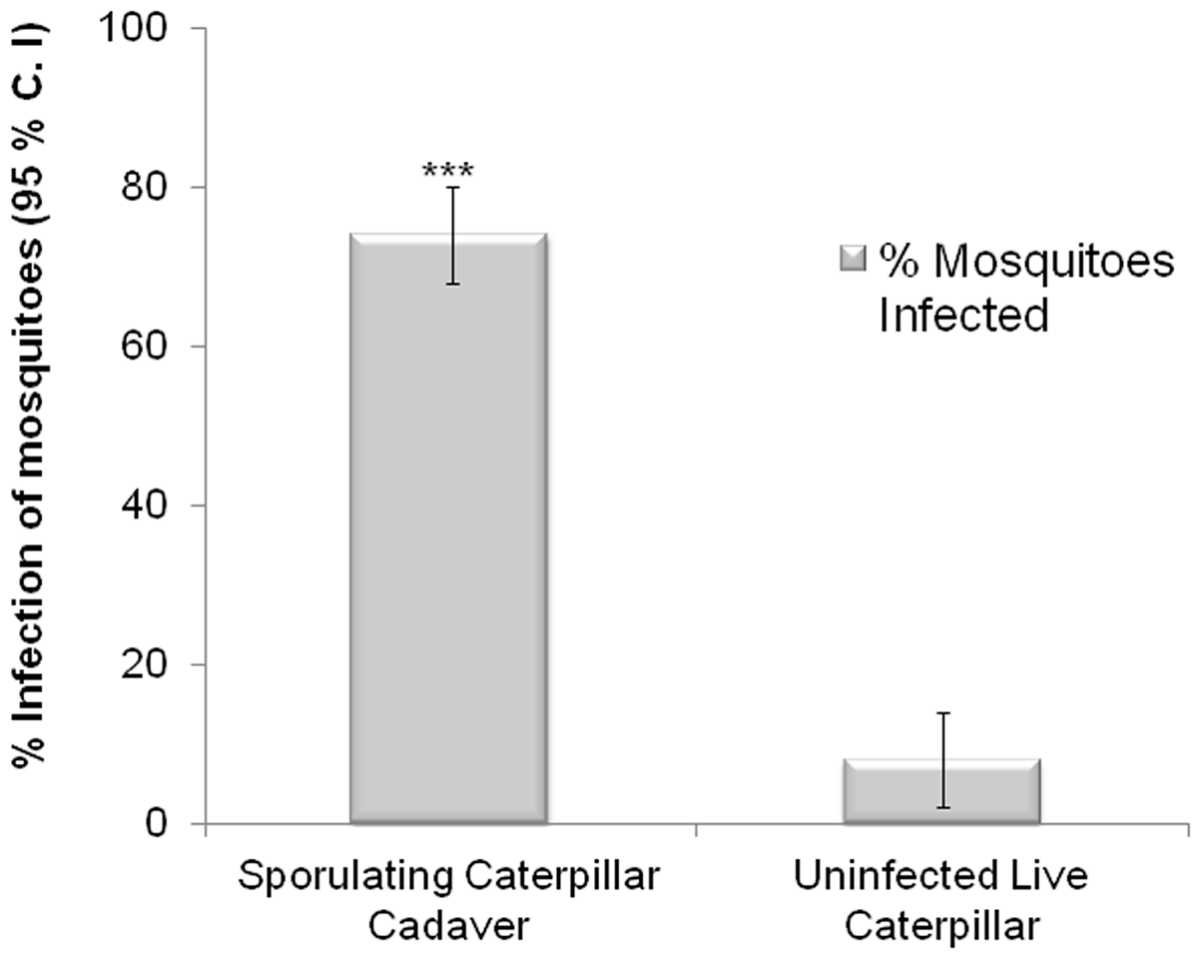
***An. stephensi*** females become infected with *B. bassiana* following contact with spore-laden caterpillars.74% (34/46) of mosquitoes contacting the sporulating caterpillars vs. 8% (4/47) contacting the uninfected caterpillars exhibited *B. bassiana* sporulation after death. N = 46 and 47 females out of 60 tested that contacted the sporulating vs. the healthy-live caterpillars, respectively. Brackets denote ±95% binomial confidence intervals (C.I.). Asterisks indicate a significant difference of infection (***P≤0.001, Chi-square 2×2 test of independence).

We released individual *An. stephensi* females in the Y-tube and observed them for their attraction and landing on or near sporulating *H. subflexa* caterpillars, captured them, and put each female into an individual sealed, humid chamber until she died, subsequently observing all cadavers over many days for signs of sporulation. The same protocol was used in a second Y-tube but instead using live, uninfected *H. subflexa* caterpillars. Of the 77% (46/60) of the females that had been attracted to the arm of the Y-tube containing sporulating caterpillars, 74% (34/46) exhibited *B. bassiana* spores within 7 days of their death. Conversely, only 8% (4/47) of the cadavers of females that had been attracted to uninfected *H. subflexa* caterpillars showed sporulation with *B. bassiana* spores 7 days post-mortem (Fig. 4).

### Sporulation Assays: Attraction of *An. stephensi* Females to Infected but Non-sporulating Caterpillars

**Figure 5:**
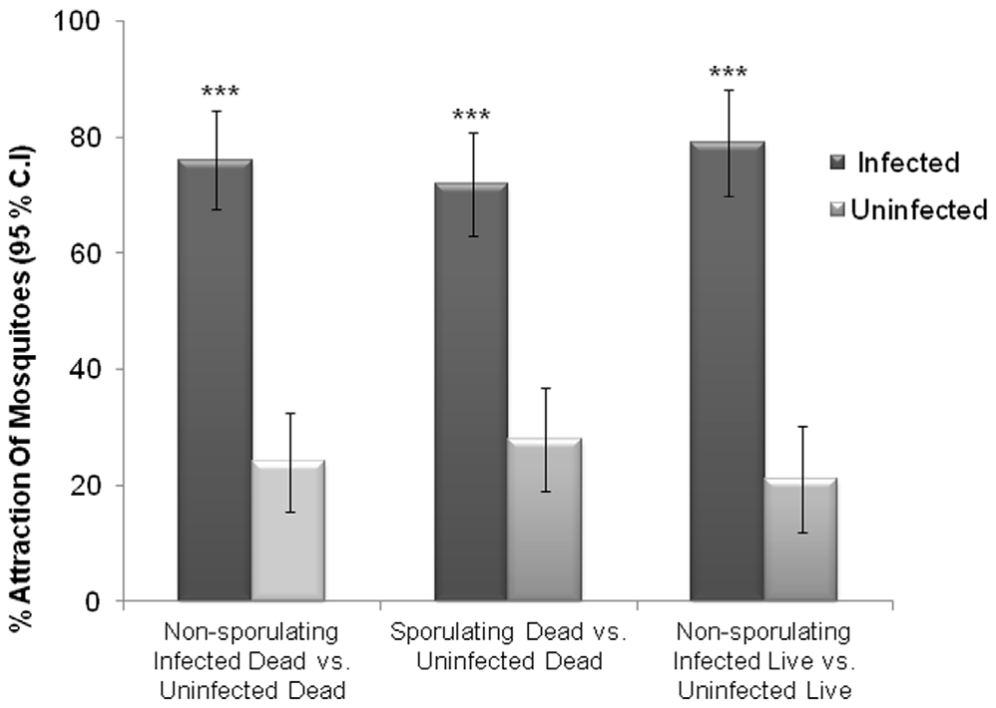
*An. stephensi* females are significantly more attracted in Y-tube choice experiments to *B. bassiana*-infected caterpillars than to uninfected caterpillars, whether they are alive or dead, sporulating or non-sporulating. (Left) percentage of female *An. stephensi* flying upwind to cadavers of 4^th^-instar *H. subflexa* caterpillars that were infected with *B. bassiana* but not sporulating (dark bars) or were uninfected (light bars). (Center) percentage of females flying upwind to cadavers of *H. subflexa* caterpillars that either exhibited *B. bassiana* spores (dark bars) or were uninfected (light bars). (Right) percentage flying upwind to *B. bassiana*-infected but non-sporulating live *H. subflexa* caterpillars (dark bars) compared to uninfected live caterpillars (light bars). (***P<0.001; Chi-square 2×2 test of independence; N = 90).

**Figure 6:**
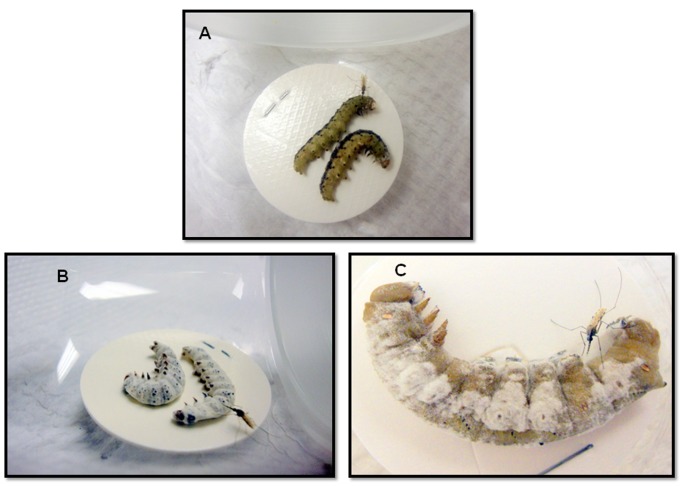
Photographs of *An. stephensi* females on sporulating and non-sporulating caterpillar cadavers after having flown upwind to them in the Y-tube olfactometer assays: A) female probing infected non-sporulating cadavers of *Heliothis subflexa* (B) female probing infected sporulating cadavers of *Heliothis subflexa* C) female probing infected sporulating cadaver of *Manduca sexta.*

We tested whether live *H. subflexa* caterpillars that had been infected with *B. bassiana* but showed no visible signs of infection would be more attractive to *An. stephensi* females than healthy, uninfected caterpillars. Surprisingly, we found that 79% of *An. stephensi* females were attracted at significantly higher levels (χ^2^ = 15.02, d.f = 1, P<0.0001) to the Y-tube arm containing healthy-looking, non-sporulating living *H. subflexa* caterpillars that had been infected with *B. bassiana* than to the arm containing uninfected living *H. subflexa* caterpillars (21%) (Fig. 5, Right). We also compared *An. stephensi* attraction to dead but non-sporulating *B. bassiana*-infected caterpillars (Fig. 6A) vs. uninfected caterpillars, both of which had been killed by freezing, then thawing them to room temperature for several hours. Seventy-six percent of *An. stephensi* females again were significantly more attracted to the Y-tube arm containing *B. bassiana*-infected non-sporulating cadavers (χ^2^ = 23.5, d.f = 1, P<0.0001) than to the arm containing uninfected cadavers (24%) (Fig. 5, Left). These levels of preference by the females were similar to that when they were made to choose between sporulating, *B. bassiana*-infected cadavers (Figs. 6B, 6C) vs. uninfected cadavers (72% vs. 28%) (χ^2^ = 17.7, d.f = 1, P<0.0001) (Fig. 5, Center).

### Respirometer Assays to Measure the CO_2_ Output from Uninfected and *B. bassiana*-Infected Caterpillars

**Figure 7:**
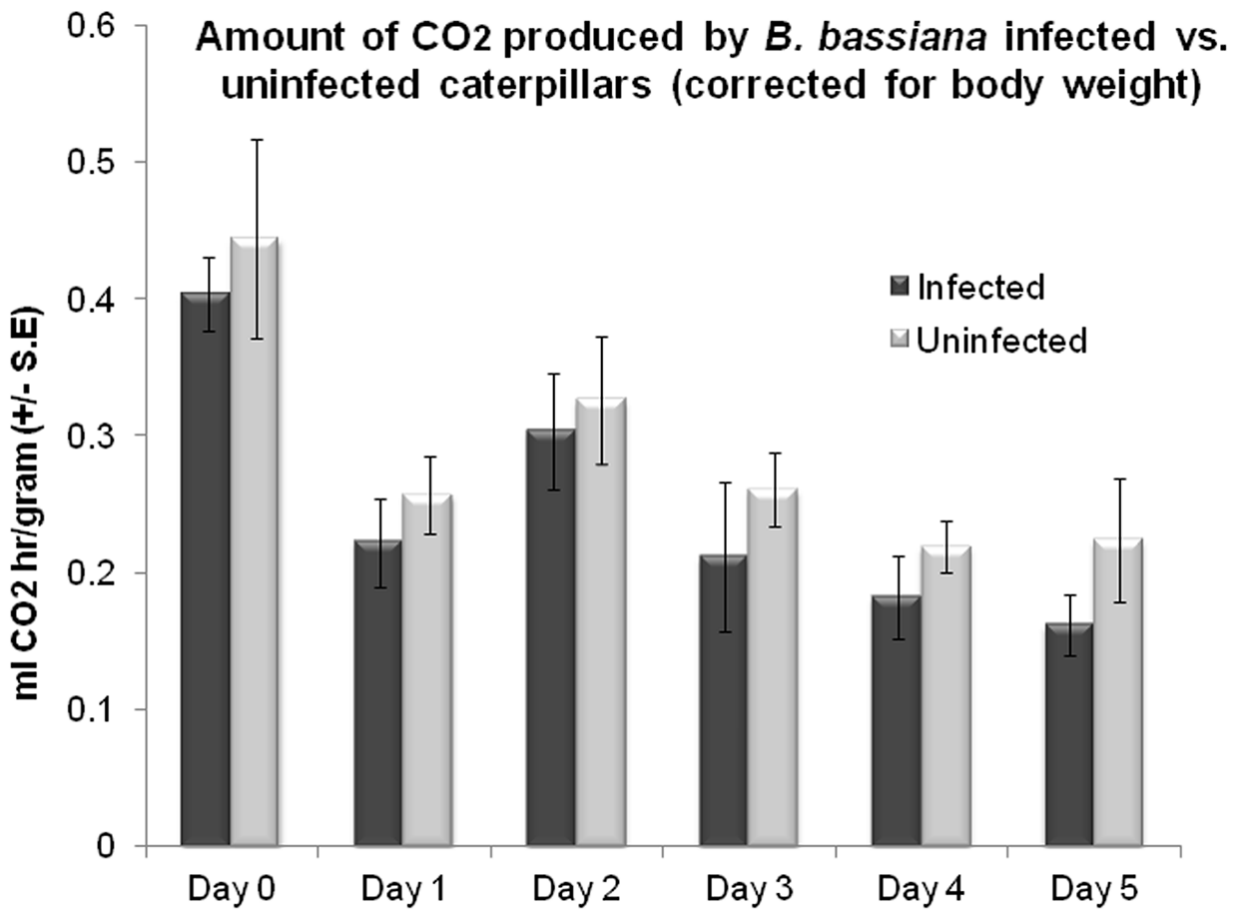
Basal Metabolic Rate (BMR) measurements showing the mean (± S.E.) amount of CO_2_ (ml CO_2_/hr/gram of body weight) produced by infected and uninfected *Heliothis subflexa* fourth instar caterpillars during the days after infection, before they were killed by *Beauveria bassiana* infection. GLM was used to examine the difference between treatments (Uninfected and *Beauveria* infected, N = 7).

There is apparently a difference between the volatiles emanating from infected caterpillars compared to uninfected ones, whether they are living or dead, sporulating or non-sporulating. However, a volatile such as CO_2_ that is known to be involved in mosquito attraction [30], [31] and is emitted by living caterpillars would not seem to be one that would explain some of the differences in *An. stephensi* attraction. For instance, both the infected non-sporulating and uninfected dead caterpillars shown in Figure 5-Left, should be emitting similar, negligible levels of CO_2_. However, it seemed possible that the living, healthy-looking infected caterpillars in Fig. 5-Right, might be emitting more CO_2_ than the live, uninfected caterpillars, but we found no significant difference in the amount of CO_2_ produced by individual caterpillars from these two groups (Fig. 7). Day factor (*F*_5, 12_ = 4.27, *P = *0.0183) was significant and there was no significant effect of treatment (*F*_1, 12_ = 1.38, *P = *0.262). The day* treatment interaction was also not significant (*F*_5, 12_ = 0.03, *P = *0.9996). Other volatiles would seem to be involved to explain this preferential attraction to infected but healthy-looking caterpillars.

### Infection of *An. stephensi* Females Following Attraction to Field-formulated *B. bassiana* Spores

**Figure 8:**
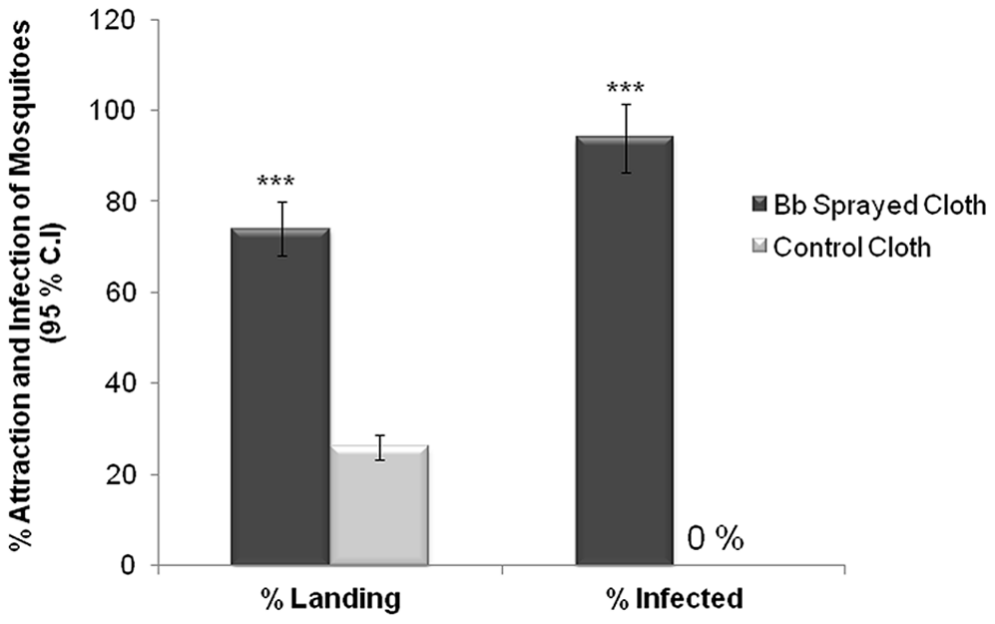
Cloth sprayed with oil-formulated spores of *B. bassiana* causes *An. stephensi* females to fly upwind and land on it (Left; dark bars), more than on an oil-only-sprayed cloth (Left; light bars). Females collected from the *B. bassiana*-sprayed cloth following a 1-minute contact duration exhibited a significantly higher rate of *B. bassiana* infection (95%) than did females allowed a 1-minute contact period (0%) with the oil-only-sprayed cloth. Brackets denote ±95% binomial confidence intervals. Asterisks indicate significant differences between landing on, and infection by, the treatment choice-pairs (Chi-square 2×2 test of independence; ***P<0.001; N = 95 mosquitoes tested for each treatment).

Finally, we investigated the attractiveness to female *An. stephensi* of field-formulated *B. bassiana* spores with regard to their potential use as a biopesticide for malaria vector control. Seventy-three percent (69/95) of *An. stephensi* females were attracted to the arm containing the *B. bassiana* spore-sprayed cloth with the remainder (27%) flying to the clean oil-sprayed cloth (Fig. 8). The mosquitoes that landed on the cloth were individually collected via aspiration after one minute of cloth exposure and provided with sugar water in individual wells of a culture plate until they died. After several days of incubation, 95% (66/69) of the females landing on the *B. bassiana*-sprayed cloth exhibited *B. bassiana* spores, whereas none of the females collected from the oil-sprayed-only control cloth showed any fungal infection (Fig. 8). These results show that at this formulated dose, *An. stephensi* females are preferentially attracted to a spore-formulation-sprayed surface and can acquire an infection after only a short exposure period.

## Discussion

Unlike most insect pathogens, fungal entomopathogens infect through contact, with fungal spores (conidia) stimulated to germinate and penetrate the insect cuticle upon contact with an appropriate host [10]. In order to utilize fungi in biopesticides it is necessary for the target insect to pick up sufficient spores either through direct application (e.g. via spraying) or through residual exposure to treated surfaces. Studies by Mburu et al. (2009) [32] showed that termites can detect the presence of *Metarhizium anisopliae* through olfaction and thus avoid direct physical contact with the fungus. Follow-on research showed that the spores of *Metarhizium* contain certain volatiles that are repellent to termites and which can be detected from a distance [33]. These results contrast with those of the current study where we find that rather than being deterred by fungal entomopathogens, *An. stephensi* females are in fact attracted to them, either as dried spores, spores formulated in oil and applied to cloth, or when the spores are present on caterpillar or mosquito cadavers. Further, even pre-lethally infected caterpillars were more attractive than uninfected caterpillars.

Why *An. stephensi* females are attracted to a lethal fungal pathogen is unclear. The volatile profiles of spores and infected caterpillars might comprise generic cues that are attractive to these mosquitoes. We did measure CO_2_ output from the caterpillars but found no effects of fungal infection on CO_2_ emission. *An. stephensi* is responsive to 1-octen-3-ol, which, in addition to being a mammalian odorant, is a well-known component of mushroom volatiles. Whether entomopathogenic fungi produce this odorant is unclear. We did conduct some exploratory GC/MS analyses of volatiles collected from dried *B. bassiana* spores using Solid Phase Microextraction (SPME) fibers but the results are too preliminary to draw any conclusions.

At a more proximate level, it is possible that mosquitoes are attracted to spores because they are associated with insect hosts that, due to fungal infection, represent an ‘easy’ food resource (reduced defensive behaviors, for example). It has been known since Harris et al.’s 1969 paper [28] that mosquito species from several genera will feed on soft-bodied insect larvae such as caterpillars and that this might have been their ancestral feeding trait [34]. However, the degree to which this behavior occurs in the field remains largely unexplored.

An alternative possibility is parasite manipulation, with the fungus attracting flying mosquito hosts to facilitate its dispersal. Hyphomycete fungi such as *B. bassiana*, which are often soil-borne, have no active mechanisms of dispersal other than via infected hosts. In some systems fungi actively manipulate behavior of infected hosts to enhance transmission (reviewed in Ref. 23). Classic examples include ‘summit disease’, whereby infected hosts climb to the top of vegetation just before death to maximize spore dispersal (e.g. grasshopper hosts infected with the fungus *Entomophaga grylli* [35]) and manipulation of biting behavior to attach infected hosts to vegetation in optimum transmission settings (e.g. leaf cutter ants infected with certain species of *Ophiocordyceps* fungi [36]). There is also evidence (albeit slightly mixed) that malaria parasites can manipulate mosquito behavior to maximize the probability of transmission [37]. Given these examples, the data we present here could fit with a manipulation hypothesis. However, natural fungal infection of adult Anopheline mosquitoes appears relatively rare [38] suggesting only a weak co-evolutionary relationship. Furthermore, we are aware of no examples of fungi manipulating uninfected host behavior via olfactory signals to facilitate transmission. Thus, while the possibility remains, it seems unlikely that the behavioral attraction we observe in the current study is an example of manipulation.

Olfaction mediated behavior plays an important role throughout the adult mosquito lifecycle, affecting host-seeking, sugar feeding, oviposition and possibly adult resting site selection [39]. In principle fungal biopesticides could be targeted at each of these points in the life cycle, with spores applied within houses to walls or hanging materials [5], [40], netting [4], eaves curtains [40], indoor or outdoor resting habitats [41], oviposition traps [42], and possibly even sugar baits [43]. Whatever the target, effective dose transfer requires sufficient residual contact with spores. In this regard, the apparent maladaptive behavioral response of malaria mosquitoes to be attracted to spores is an unexpected benefit that could increase the vector control potential of the biopesticide approach.

## Materials and Methods

### Mosquito Rearing

*Anopheles stephensi* Liston were reared under standard insectary conditions of 27°C, 80% humidity and 12∶12 light: dark photo-period. Eggs were placed in plastic trays (25 cm×25 cm×7 cm) filled with 1.5 l of distilled water. To reduce variation in adult size at emergence, larvae were reared at a fixed density of 400 per tray. Larvae were fed Liquifry for five days and then on Tetrafin fish flakes. From approximately two weeks after egg hatch, pupae were collected daily and placed in emergence cages. The adults that emerged were fed *ad libitum* on a 10% glucose solution.

### Production of Fungal Spores

#### Liquid Culture

Conidia were harvested from slopes or plates to make a spore suspension of approximately 1×10^6^ conidia ml^−1^ in sterile 0.05% w/v Tween 80 (Sigma) in distilled water. One ml of this suspension was then used to inoculate 75 ml sterile liquid medium culture medium (4% d-Glucose, 2% yeast extract [Oxoid, UK] in tap water), in 250 ml Erlenmeyer flasks. Flasks were incubated on a rotary shaker (160 rpm) at 24°C for 3 days.

#### Solid substrate

Barley flakes (Bobs Red Mill, Milwaukie, Oregon, USA) were weighed into mushroom spawn bags (Unicorn, Garland, Texas, USA), 1 kg per bag and 600 ml tap water was added and the contents mixed by hand to ensure even absorption of the water. The spawn bags were then placed inside autoclave bags for protection and autoclaved for 30 min at 121°C [44]. Once cool the bags were inoculated under aseptic conditions with 75 ml of the 4 day old liquid medium plus 75 ml of sterile water to achieve a final moisture content of approximately 48%. The inoculated bags were carefully massaged to ensure even distribution of the inoculum. The bags were then sealed and incubated on shelves for 10 days at 24°C. Following incubation, the bags were opened in a reverse flow cabinet (Labconco, USA) and the contents transferred to brown paper bags for drying. The paper bags were placed in a dehumidified room for 4 days (24–30°C), until the sporulated substrate reached <20% moisture content. The conidia were then harvested from the barley flakes using a Mycoharvester (Acis Manufacturing, Devon, UK). The harvested conidia were placed in glass dishes and further dried in a desiccator over dry silica gel at 24°C. Once the conidia powder reached 5% moisture content, a small sample was taken for quality analysis and the remaining powder was sealed in foil laminated sachets and stored at 5°C until use. The *Beauveria bassiana* isolate used was I93–825 and the *Metarhizium anisopliae* isolate used was IMI330189.

### Olfactometer Assays

All behavioral experiments used four-to six-day-old adult *An. stephensi* females. The sugar solution used during their holding period as adults was removed four hours before they were used in the experiments. Behavioral assays employed a Y-tube olfactometer (70 cm×35 cm×6 cm) to examine the upwind flight behavior of mosquitoes towards various potentially attractive sources. All assays were performed at mid-afternoon in the mosquitoes’ daily light cycle and under an ambient light intensity of 630 Lux. In all experiments, one female *An. stephensi* mosquito was released at the main (common) downwind end of the Y-tube, with charcoal purified air flowing equally through both arms of the tube at a rate of 0.75 liter/sec. Each mosquito was observed until it flew upwind and settled at the end of one of the two upwind arms. Each mosquito was used only once in the experiment and subsequently killed via freezing. For all of the assays, the arms of the Y-tube were flipped 180° after 15 individual flights to avoid any possible lighting-related or other unknown bias, and the interior walls of the entire apparatus were rinsed with hexane at that time. Percentage responses in each upwind movement category were analyzed for each experiment using a Chi-square test of independence with Yates’ correction.

### Attraction of *An. stephensi* Females to Dried Spores of Various Fungal Species

Fifty milligrams of the dried spores of either *Beauveria bassiana, Metarhizium anisopliae,* or *Penicillium* spp. were placed on a small cardboard disc (2 cm diameter) and inserted at the upwind end of one of the two arms of the Y-tube. The alternate, control, arm contained a blank cardboard disc. In the choice assays testing *B. bassiana* against other fungal spores, *B. bassiana* spores were placed on a cardboard disc in one arm and the other fungal spores were placed on a cardboard disc in the other arm. Percentage responses in each upwind movement category were analyzed for each day post-infection using Chi-square test of independence with Yates’ correction (N = 60).

### Attraction of *An. stephensi* Females to Sporulating Insect Cadavers

Fourth instar larvae of *M. sexta* and *H. subflexa* were infected with *B. bassiana* by dusting them with spores. Before their death after ca. 5–7 days, examination of their cuticle under a light microscope revealed a clean cuticle with no evidence of residual spores from the spore-dusting a week prior. The caterpillars subsequently died of infection and sporulated within the next week. To test the behavior of *An. stephensi* towards sporulating vs. uninfected cadavers, females (N = 60) were released at the main end of the Y-tube and their choice preferences were recorded. *An. stephensi* females were infected by attracting them to fungal spores on a treated surface, letting them have contact with the spores for two minutes, and then allowing them to die from fungal infection and to sporulate in a sealed petri dish under moist conditions. Uninfected females that had died for other reasons, including age or dessication, were used as controls. The sporulating females were placed inside one arm of the Y-tube and the uninfected dead mosquitoes were placed in the other arm. Sixty mosquitoes were flown in the olfactometer assays.

Y-tube assays were performed pitting sporulating cadavers against freeze-killed uninfected fourth instar caterpillars of the same species in the alternate upwind arm. The freeze-killed caterpillars had previously been warmed to room temperature for two hours before being placed in the olfactometer arm. The attraction of *An. stephensi* females to the cadavers of *H. subflexa* caterpillars that had been infected with *B. bassiana* but showing no visible signs of infection was performed by using fourth-instar caterpillars after four days post-infection. Freeze-killed uninfected fourth-instar *H. subflexa* caterpillars were used as controls, and the freeze-killed caterpillars were warmed for two hours at room temperature before commencement of the assays. Attraction to non-sporulating, four-day post-infection *B. bassiana*-infected living *H. subflexa* caterpillars was assessed compared to their attraction to living, uninfected *H. subflexa* caterpillars in the alternate Y-tube arm. Ninety mosquitoes were used in each olfactometer assay.

### Sporulation Assays

The Y-tube olfactometer assays that were performed assessed the *An. stephensi* females’ ability to fly upwind and become infected by landing on or near the sporulating caterpillar cadavers of *H. subflexa.* Individual mosquitoes (N = 60) that landed on or near the sporulating caterpillar cadavers at the upwind arm of the Y-tube were gently picked up using a clean aspirator and each female was put into an individually sealed, petri-well-plate chamber containing a moistened filter paper. The wells were sealed using paraffin film and after death, the mosquitoes were checked daily for evidence for spores of *B. bassiana,* the identification being confirmed under light-microscopy. The mosquitoes that flew to the other Y-tube arm were also collected, incubated in individual petri-well-plates and observed daily once they had died. The same protocol was used in a second Y-tube, but instead using live, uninfected *H. subflexa* caterpillars.

### Respirometer Assays to Measure the CO_2_ Output from Uninfected and *B. bassiana*-Infected Caterpillars

Previous studies have shown that mosquitoes that are infected with *B. bassiana* fungus increase their CO_2_ output during the days following infection before they are killed by the fungus [5]. Therefore, we wanted to determine whether this increase in CO_2_ output resulting from fungal infection might be a factor in mosquitoes’ attraction to infected caterpillars. Early fourth instar larvae of *H. subflexa* were infected by pipetting 5 µl of liquid formulation containing 10,000 spores/µl. We measured the amount of CO_2_ produced by infected-live and uninfected healthy-live caterpillars for 5 days after infection or until they died due to infection. Metabolic rates were measured by placing each infected or uninfected caterpillar in an individual flow-through respirometry chamber [5]. Dry CO_2_-free air was passed through the 20 ml chambers at 0.25 litres/min and then dried and passed through a Li-Cor 6252 CO_2_ analyser. Within each run, 7 experimental chambers containing single caterpillars were sampled in sequential fashion by using a computer-controlled valve system. Three chambers contained fungus-infected live caterpillars and four chambers contained uninfected live caterpillars in the first run, and four infected vs. three uninfected caterpillars were assessed in the second runs, giving 7 replicates per day of each caterpillar type. An eighth chamber was left empty and sampled at the same time as each of the occupied chambers to establish a baseline. All chambers were housed in a reach-in incubator set to 25° (±0.2°) C. Analog signals from the flow meter and CO_2_ analyzer were converted to digital and recorded on a computer (Sable systems, Salt Lake City). The caterpillars were used only once in each trial. Two-way ANOVA was performed using GLM procedures to analyze the data.

### Infection of *An. stephensi* Females Following Attraction to Field-formulated *B. bassiana* Spores

The attractiveness to female *An. stephensi* of field-formulated *B. bassiana* spores with regard to their potential use as a biopesticide for malaria vector control was tested using Y-tube olfactometer assays. Oil-formulated *B. bassiana* spores [2] were sprayed onto a white polyester mesh cloth that was stretched over the end of one arm of the Y-tube olfactometer. An oil-only sprayed cloth was stretched over the end of the other Y-tube arm as a control. The behavior of mosquitoes (N = 95) were tested as explained earlier in the olfactometer assays, and the mosquitoes that landed on the cloth were individually collected via aspiration after one minute of cloth exposure and provided with sugar water in individual wells of a culture plate until they died. The mosquitoes were incubated and checked for mycelial growth resulting from *B. bassiana* infection.
